# Increased nocturnal urinary cortisol levels in the elderly patients with depression, coexisting major geriatric syndromes and combined pathogenetic mechanisms

**DOI:** 10.1007/s40520-024-02849-w

**Published:** 2024-09-27

**Authors:** Antonio Martocchia, Manuela Stefanelli, Maurizio Gallucci, Marianna Noale, Stefania Maggi, Maurizio Cassol, Demetrio Postacchini, Antonella Proietti, Mario Barbagallo, Ligia J. Dominguez, Claudio Ferri, Giovambattista Desideri, Lavinia Toussan, Francesca Pastore, Giulia M. Falaschi, Giuseppe Paolisso, Paolo Falaschi, The AGICO Investigators, Antonio Martocchia, Manuela Stefanelli, Maurizio Gallucci, Maurizio Cassol, Demetrio Postacchini, Mario Barbagallo, Claudio Ferri, Giovambattista Desideri, Lavinia Toussan, Francesca Pastore, Giuseppe Paolisso, Paolo Falaschi, Stefano Eleuteri, Giulia Maria Falaschi, Maria Grazia Oddo, Cinzia Giuli, Ilenia Macchiati, Monica Migale, Francesca Sorvillo, Roberto Brunelli, Monia Francavilla, Silvia Santini, Luciano Marini, Elpidio Santillo, Luca Fallavollita, Sara Rotunno, Michelangela Barbieri, Edith Angellotti, Valeria Ludovici, Paola Cheli, Rita Del Pinto, Ligia J Dominguez, Giovanna Bella, Valentino Culotta

**Affiliations:** 1 S. Andrea Hospital, Sapienza University of Rome, Via Di Grottarossa 1035, 00189 Rome, Italy; 2 Casa Di Cura Villa Domelia, Rome, Italy; 3 Cognitive Impairment Centre, Local Unit of Health and Social Services N.2, Marca Trevigiana, Treviso, Italy; 4 Aging Branch, Neuroscience Institute, National Research Council (CNR), Padua, Italy; 5 S. Pietro Fatebenefratelli Hospital, Rome, Italy; 6 Geriatrics Operative Unit, Italian National Research Centre On Aging (IRCCS INRCA), Fermo, Italy; 7 Department of Medicine, Geriatric Unit, University of Palermo, Palermo, Italy; 8 Department of Clinical Medicine, Life, Health and Environmental Sciences, University of L’Aquila, L’Aquila, Italy; 9 Department of Clinical, Internal Medicine, Anesthesiologic and Cardiovascular Sciences, Sapienza University of Rome, Rome, Italy; 10 RSA Anni Azzurri Parco Di Veio, Rome, Italy; 11 Kos Care Villa Margherita, Benevento, Italy; 12 Department of Advanced Medical and Surgical Sciences, University of Campania “Luigi Vanvitelli”, Naples, Italy

**Keywords:** Cortisol, Aging, Depression, Dementia, Metabolic syndrome

## Abstract

**Background:**

The mechanisms at the basis of depression are still matter of debate, but several studies in the literature suggest common pathways with dementia (genetic predispositions, metabolic and inflammatory mechanisms, neuropathological changes) and other geriatric syndromes.

**Aims:**

To evaluate the role of cortisol (as marker of the HPA, hypothalamus–pituitary–adrenal axis hyperactivity) in elderly subjects with depressive symptoms (by the means of the AGICO, AGIng and COrtisol, study), in relationship to the presence of the major geriatric syndromes.

**Methods:**

The AGICO study enrolled patients from ten Geriatric Units in Italy. Every subject received a comprehensive geriatric assessment or CGA (including the Mini Mental State Examination or MMSE, Geriatric Depression Scale or GDS and Cornell Scale for Depression in Dementia or CSDD), the neurological examination (with a computed tomography scan or magnetic resonance imaging of the brain), the assessment of the metabolic syndrome (MetS), the evaluation of the cortisol activity by two consecutive urine collections (diurnal and nocturnal), a CGA-derived frailty index (FI) and a modified measure of allostatic load (AL).

**Results:**

The MMSE scores were significantly and inversely related to the values of GDS (p < 0.001) and CSDD (p < 0.05), respectively. The patients with depressive symptoms (GDS/CSDD > 8) showed significantly increased disability, MetS, inflammation, FI and AL and significantly reduced MMSE and renal function.

The diurnal and nocturnal urinary cortisol levels in the patients with depressive symptoms (GDS/CSDD > 8) were higher with respects to controls (p < 0.05 for nocturnal difference).

**Discussion:**

The AGICO study showed that the stress response is activated in the patients with depression.

**Conclusion:**

The depression in elderly patient should be reconsidered as a systemic disease, with coexisting major geriatric syndromes (disability, dementia, frailty) and combined pathogenetic mechanisms (metabolic syndrome, impaired renal function, low-grade inflammation, and allostatic load). Cortisol confirmed its role as principal mediator of the aging process in both dementia and metabolic syndrome.

## Introduction

Depression and dementia are the bad companions in the elderly patients. The mechanisms at the basis of their relationship are still matter of debate, but several studies in the literature suggest common genetic predispositions, metabolic and inflammatory pathways, and neuropathological changes [1–5]. From a bidirectional point of view, the risk of developing dementia in the patients with depression is > 50% (up to twofold increase) [6–11], and the presence of the depressive symptoms in the patients with dementia is about 40–50% [11–14].

The allostatic load (AL), the metabolic syndrome (MetS) and the neuroendocrine activation of the stress cascade (with particular regard to the hypothalamus–pituitary–adrenal HPA axis) are the major risk factors for both depression and dementia [15–21].

The national multicenter observational AGICO (AGIng and COrtisol) study recently examined the role of cortisol in elderly subjects, showing a trend to increased cortisol excretions in elderly patients with MetS, as a marker of the HPA axis hyperactivity induced by the peripheral mechanisms and inflammation [22]. The AGICO study also demonstrated an increase of both diurnal and nocturnal urinary cortisol in dementia (by central mechanisms), with the highest levels in patients with associated dementia and MetS (increase of about 20% and 26%, respectively) [23].

The aim of the present study is to evaluate the role of cortisol (as marker of the HPA axis hyperactivity) in elderly subjects with depressive symptoms, in relationship to the presence of the major geriatric syndromes, by the cortisol measures from urine samples using the national multicenter AGICO study.

## Materials and methods

The AGICO Study was approved by the ethics committee of Sapienza University of Rome (Prot CE 372/2011, Study Coordinator Prof. Paolo Falaschi) [22]. In brief, the AGICO Study enrolled patients from ten Geriatric Units in Italy (the detailed list in the “Appendix”) in 2012–2017, after an informed consent by participants or their legally authorized representatives. Exclusion criteria were exogenous hypercortisolism (by systemic glucocorticoid therapy) and adrenal gland diseases (like Cushing’s syndrome or Addison’s disease). Every subject received a comprehensive geriatric assessment (CGA), including the Activity of Daily Living (ADL), Instrumental Activity of Daily Living (IADL), Cumulative-Illness Rating Scale (CIRS) -severity (-SI) and -comorbidity index (-CI), Mini-Mental State Examination (MMSE), Geriatric Depression Scale (GDS) and Cornell Scale for Depression in Dementia (CSDD) [24–29]. The neurocognitive disorders (depression and dementia) were examined according to the Diagnostic and Statistical Manual of Mental Disorders fifth edition (DSM-5) criteria and the International Classification of Diseases tenth revision (ICD-10) [30]. A complete neuropsychological examination was performed: a MMSE cutoff value of 24 was used for the diagnosis of dementia and a GDS/CSDD score ≥ 8 for the diagnosis of depression [31, 32]. The neurological examination included a computed tomography (CT) scan or magnetic resonance imaging (MRI) of the brain (for differential diagnosis of dementia), with a brain atrophy score (BAS) (0–3) and vascular brain injury score (VBIS) (0–3) [22]. The investigators assessed clinical data, anthropometric (with the body mass index = BMI) and cardio-metabolic measurements [22]. For the assessment of the metabolic syndrome (MetS), the Adult Treatment Panel III (ATP III) criteria was used (≥ 3 factors, among abdominal obesity, hypertriglyceridemia, low HDL cholesterol, elevated blood pressure and impaired fasting glucose) [22]. Every patient collected two consecutive urinary samples of 12 h (from 8 a.m. to 8 p.m. during the day, and from 8 p.m. to 8 a.m. during the night), for the measurement of the cortisol activity and urinary creatinine (by immunochemiluminescent method of Immulite 2000 Cortisol, Siemens Healthcare Diagnostic Products Ltd. Llanberis, Gwynedd LL55-4EL, United Kingdom; normal values 70–320 μg/24 h, total imprecision < 8% CV). The results of the urinary cortisol were reported as μg/g urinary creatinine (to adjust for body size and renal function). As previously described, the nocturnal sample minimized the variation in the cortisol excretion due to the daytime physical activity.

A frailty index (FI) was derived by the CGA, based on the previously described FI-CGA of Jones et al. [33], evaluating the sum of the ADL (0 = independent, 0.5 = dependence in ADL ≤ 2, 1 = dependence in ADL > 2), IADL (0 = independent, 0.5 = dependence in IADL ≤ 3 in females and ≤ 2 in males, 1 = dependence in IADL > 3 in females and > 2 in males), cognition (0 = no cognitive impairment MMSE ≥ 27, 0.5 = cognitive impairment and no dementia 24 ≤ MMSE < 27, 1.0 = dementia with MMSE < 24), emotion (0 = GDS/CSDD < 5, 0.5 = 5 ≤ GDS/CSDD ≤ 10, 1 = GDS/CSDD > 10), comorbidity (CIRS-SI value, range 1–5), malnutrition (0 = BMI ≥ 23, 0.5 = 23 < BMI ≤ 19, 1 = BMI < 19) (FI range = 0/0–10/10 = 0–1).

A modified measure of allostatic load (AL) was calculated for each participant, as an index of dysregulated homeostatic systems, when the biomarkers exceed the cut-off levels, such as the presence of MetS (0–5), inflammation (C-reactive protein CRP > 3 mg/dl), reduced kidney function (creatinine clearance Cr-clear < 60 ml/min, measured by Chronic Kidney Disease Epidemiology Collaboration creatinine equation) and HPA axis activation (nocturnal urinary cortisol > 25.7 μg/g urinary creatinine) (AL range 0/8–8/8 = 0–1) [33–35].

Data have been presented as mean ± standard deviation (m ± SD).

The statistical analyses were performed using the Kolmogorov–Smirnov test (to evaluate the normal distribution of the data), the one-way analysis of variance (ANOVA), the Mann–Whitney test for the comparison of data between independent groups (diurnal and nocturnal cortisol levels), the Wilcoxon signed-rank test to compare two groups using a set of matched samples (differences between the diurnal and nocturnal cortisol levels in the single patient), the linear regression models for the associations between the variables (age, MMSE, GDS, CSDD and cortisol). A value of p < 0.05 was considered statistically significant.

## Results

We enrolled n.276 patients. The MMSE scores were significantly and inversely related to the values of GDS (r = -0.230 p < 0.001) and CSDD (r = -0.308 p < 0.05), respectively. The GDS and the CSDD scores were significantly and positively related (r =  + 0.870 p = 9.1 × 10^–9^); 1 GDS point approximately corresponded to 1.7 CSDD point.

The clinical features of the patients with depressive symptoms (Dep with GDS/CSDD ≥ 8, n.89) and without depressive symptoms (no-Dep with GDS/CSDD < 8, n.187), were respectively: age 79.5 ± 6.0 years, males M = 35%, ADL = 5.2 ± 1, IADL M = 3.8 ± 1.5, IADL F = 5.5 ± 2.7, BMI = 27.3 ± 4.3, CIRS-SI = 2.0 ± 0.4, CIRS-CI = 4.4 ± 1.8, MetS score = 2.7 ± 1.1, CRP = 2.7 ± 4.3 mg/dl, Cr-clear = 59.2 ± 21.9 ml/min, FI = 0.5 ± 0.1, AL = 0.6 ± 0.2; age 77.7 ± 7.3 years, M = 42%, ADL = 3.8 ± 1.9, IADL M = 1.8 ± 1.5, IADL F = 3.1 ± 2.6, BMI = 27.7 ± 4.3, CIRS-SI = 2.1 ± 0.4, CIRS-CI = 4.5 ± 1.8, MetS score = 2.3 ± 1.1, FI = 0.3 ± 0.1, CRP = 0.9 ± 1.5 mg/dl, Cr-clear = 67.6 ± 20.5 ml/min, AL = 0.5 ± 0.2 (age Dep versus no-Dep F = 10.7 p < 0.01, ADL F = 60.5 p = 1.5 × 10^–13^, IADL-M F = F = 41.0 p = 4.6 × 10^–6^, IADL-F F = 26.4 p = 8.3 × 10^–7^, MetS F = 6.9 p < 0.01, CRP F = 18.7 p = 2.5 × 10^–5^, Cr-clear F = 7.3 p < 0.01, FI F = 97.2 p = 2 × 10^–18^, AL F = 16.7 p = 5.7 × 10^–5^) (Tab. 1).

**Table 1:** The clinical characteristics of the patients with (Dep) or without (no-Dep) depressive symptoms

	Dep patients (n.89)	No-Dep patients (n.187)	Statistical analyisis	p value
Age	79.5 ± 6.0	77.7 ± 7.3	F = 10.7	p < 0.01
Males	35%	42%		
ADL	3.8 ± 1.9	5.2 ± 1.1	F = 60.5	p = 1.5 × 10^–13^
IADL M	1.8 ± 1.5	3.8 ± 1.5	F = 41.0	p = 4.6 × 10^–6^
IADL F	3.1 ± 2.6	5.5 ± 2.7	F = 26.4	p = 8.3 × 10^–7^
BMI	27.7 ± 4.3	27.3 ± 4.3		
CIRS-SI	2.1 ± 0.4	2.0 ± 0.4		
CIRS-CI	4.5 ± 1.8	4.4 ± 1.8		
MetS Score	2.7 ± 1.1	2.3 ± 1.1	F = 6.9	p < 0.01
MMSE	20.1 ± 6.9	24.5 ± 6.8	F = 37.0	p = 3.9 × 10^–9^
GDS	9.1 ± 3.3	3.3 ± 2.1	F = 275.7	p = 3.1 × 10^–42^
CSDD	13.6 ± 4.8	3.9 ± 2.4	F = 51.5	p = 4.0 × 10^–9^
FI	0.5 ± 0.1	0.3 ± 0.1	F = 97.2	p = 2 × 10^–18^
CRP	2.7 ± 4.3	0.9 ± 1.5	F = 18.7	p = 2.5 × 10^–5^
Cr-clear	59.2 ± 21.9	67.6 ± 20.5	F = 7.4	p < 0.01
AL	0.6 ± 0.2	0.5 ± 0.2	F = 16.7	p = 5.7 × 10^–5^

*ADL* activity of daily living (6/6), *IADL* instrumental activity of daily living , *M* males (5/5) and *F*  females (8/8), *CIRS* cumulative-illness rating scale , *-SI* -severity , *-CI *-comorbidity index, *BMI*  body mass index, *MetS* metabolic syndrome,* MMSE* mini-mental state examination, *GDS* geriatric depression scale , *CSDD* cornell scale for depression in dementia , *FI* frailty index, *CRP* C-reactive protein, *Cr-clear *creatinine clearance, *AL *allostatic load

The MMSE, GDS and CSDD values from the neurological examination resulted: 20.1 ± 6.9, 9.1 ± 3.3 and 13.6 ± 4.8 in the Dep group and 24.5 ± 6.8, 3.3 ± 2.1 and 3.9 ± 2.4 in the no-Dep group (Dep vs no-Dep MMSE F = 37.0 p = 3.9 × 10^–9^, GDS F = 274.7 p = 3.1 × 10^–42^, CSDD F = 51.5 p = 4.0 × 10^–9^) (Tab. 1).

**Fig. 1:**
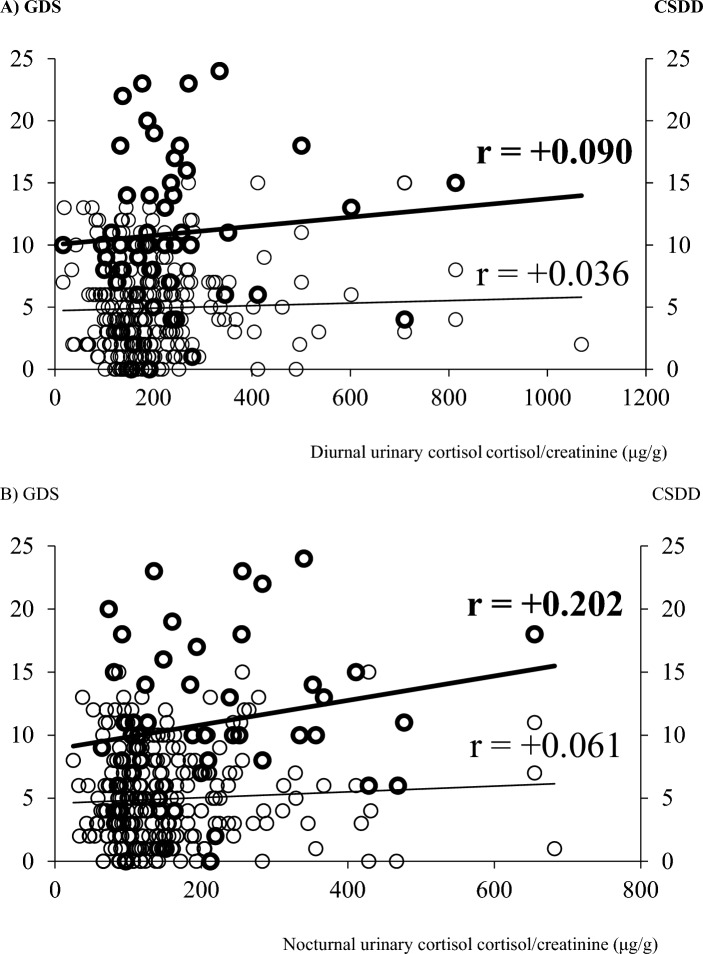
The linear regression analysis of the depressive symptoms (GDS/CSDD scores) and the diurnal** (A) **and nocturnal urinary cortisol** (B**) in Dep (bold line) and in no-Dep group. *GDS*  geriatric depression scale, *CSDD* cornell scale for depression in dementia. **A** GDS CSDD**.** Diurnal urinary cortisol cortisol/creatinine (μg/g). **B** GDS CSDD. Nocturnal urinary cortisol cortisol/creatinine (μg/g)

The depressive symptoms (GDS/CSDD scores) were positively related to the diurnal and nocturnal urinary cortisol in Dep (r =  + 0.036 and r =  + 0.061 for GDS) and in no-Dep group (r =  + 0.090 and r =  + 0.202 for CSDD), respectively (Fig. 1).

**Fig. 2:**
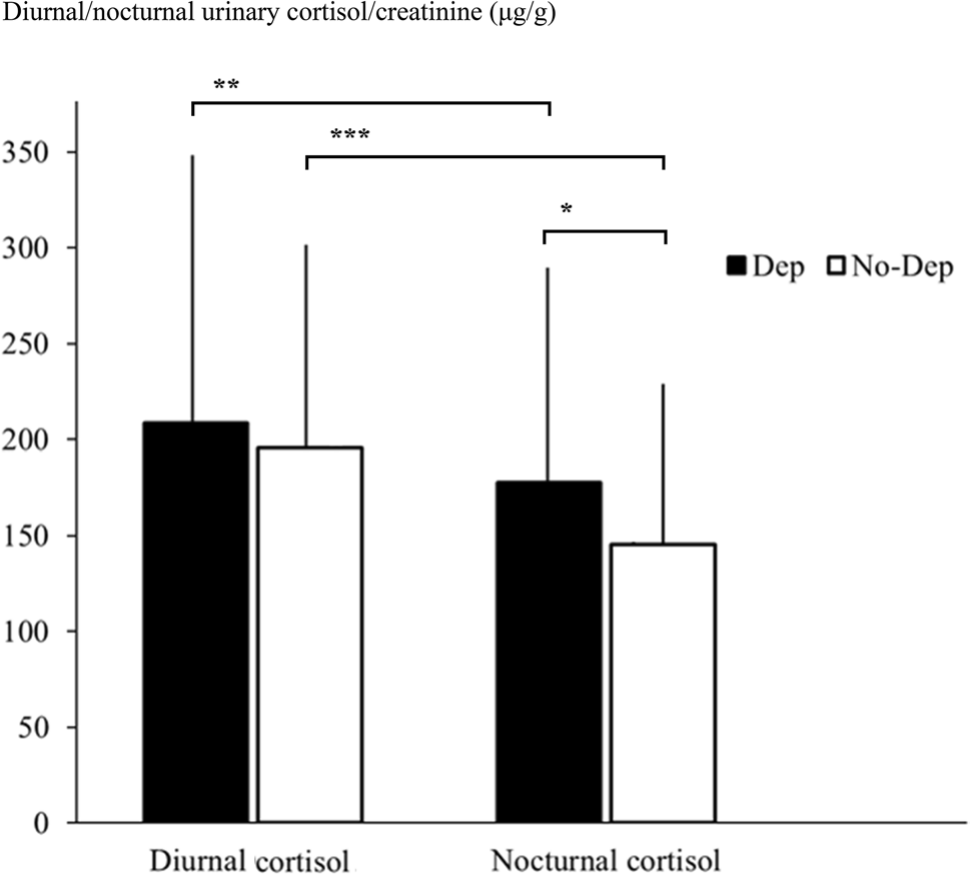
The diurnal and nocturnal urinary cortisol/creatinine (μg/g) in the Dep (n.89) and no-Dep (n.187) patients of the AGICO study (* p < 0.05, ** p < 0.025, *** p < 0.00001). Diurnal/nocturnal urinary cortisol/creatinine (μg/g)

The diurnal and nocturnal urinary cortisol levels in the Dep patients (with GDS/CSDD ≥ 8) were 208.3 ± 148.1 and 177.8 ± 117.9 μg/g and in the no-Dep patients (with GDS/CSDD < 8) were 195.7 ± 113.3 and 145.4 ± 87.4 μg/g (diurnal vs nocturnal urinary cortisol in Dep Wilcoxon 2.3 p < 0.025, diurnal vs nocturnal urinary cortisol in no-Dep Wilcoxon 8.2 p < 0.00001, nocturnal urinary cortisol in Dep vs no-Dep patients Mann–Whitney 2.2 p < 0.05) (Fig. 2).

## Discussion

The present study showed the significant increase of the nocturnal urinary cortisol in the elderly patients with depressive symptoms. These results are consistent with previous reports by other groups that found an increase of the ratio of evening/morning salivary cortisol (as a marker of HPA hyperactivity) among patients with the depression/co-morbid dementia and an increase of cerebrospinal cortisol associated with higher scores of depression after controlling for covariates.

including Alzheimer disease pathology [32, 36].

As matter of fact, depression was associated with coexisting disability (about 80% of subjects for ADL and IADL), dementia (70%), metabolic syndrome (55%), impaired renal function (30%) and low-grade inflammation (15%) in our Dep group. The indices of combined risk factors (as frailty and allostatic load) showed values as high as more or equal than half of the indices themselves (0.5 for frailty and 0.6 for allostatic load, respectively) in the Dep group.

On the contrary, all the comorbidities and risk factors were less frequent in the no-Dep group: disability (45%), dementia (40%), metabolic syndrome (45%), impaired renal function (25%), low-grade inflammation (2%), frailty (0.3) and allostatic load (0.5).

These results confirmed the study hypothesis and detailed our previous reports of the AGICO investigators examining the role of cortisol in the metabolic syndrome, dementia, low-grade inflammation and allostatic load [16, 21–23]. In particular, the elevation of cortisol in dementia has been related to central mechanisms (loss of the inhibitory feedback of the damaged hippocampus on the HPA axis function), whereas the peripheral mechanisms (hyperactivity of the β-hydroxysteroid dehydrogenase type I in the abdominal adipose tissue) has been involved in the metabolic syndrome [21–23, 37]. With regards to the allostatic load, the cortisol is the commonly used main parameter for the measurement of the activation of the HPA axis [38] and it is well-known that it is associated to low-grade inflammation and the development of the frailty syndrome in aging [22, 39]. In a study in young adult patients (mean age 33.7 years) with major depression, the authors found clinically significant high allostatic load (≥ 4) when comparing it in non-depressed controls [40], even if the results were not so clear as in our elderly patients.

More recently, the role of the allostatic load (with increased inflammation and cortisol levels) has been proposed for the development of renal failure, starting from a perturbation of the abdominal gut microbiota [41].

The deterioration of the renal failure in our Dep group (stage 3 or moderate renal failure NFK KDOQI) exceeded what expected for the difference of ages with respect to the no-Dep group (with stage 2 or mild renal failure) [42]. Shared mechanisms for the more rapid decrease in kidney function in depressed patients have been proposed, such as an autonomic imbalance activating the HPA axis, a significant inflammation with large amount of cytokine, a concomitant diffuse vascular damage con small vessel disease [43–46].

Therefore, we strongly support the evidence that the depression in elderly patient should be definitely reconceptualized as a systemic disease, beyond the classical neurochemical point of view [2, 47, 48].

When examining the association of depression and cardiovascular disease, anti-depressive treatment did not reduce the elevated cardiovascular risk (see for example the eIMPACT randomized controlled trial) [49]. A combined intervention approach against both depression and its candidate biological mechanisms should be required, with multiple treatments targeting the diet, physical inactivity, smoking, polypathology and social support [50].

Several limitations should be noted within our study, such as the presence of confounding factors (age, gender, lifestyle factors, comorbidities and medication use), but the sub-categorization should excessively shrink the sample size in each subgroup. In the present study, the gender differences and the scores for comorbidities (both CIRS-CI and CIRS-SI) were not statistically different between the sample subgroups. Regarding the polypharmacy, further longitudinal studies are necessary to evaluate the effects of specific therapies on the development of depressive symptoms in elderly subjects.

Future researches will explore whether the modulation of the cortisol levels by innovative therapies acting at the physio-pathological mechanisms (central/peripheral HPA hyperactivity) could ameliorate the depressive symptoms together with the major geriatric syndromes.

In conclusion, the present report of the AGICO study found increased cortisol levels in elderly patients with sustained depressive symptoms, as a marker of the HPA activation and with a significant role as mediator in overall geriatric syndrome dementia, allostatic load, disability and frailty.

## Data Availability

No datasets were generated or analysed during the current study.
